# Reported Adverse Drug Reactions in Infants: A Nationwide Analysis in Malaysia

**DOI:** 10.3389/fphar.2017.00030

**Published:** 2017-02-10

**Authors:** Rosliana Rosli, Ahmad Fauzi Dali, Noorizan Abd. Aziz, Long Chiau Ming, Mohamed Mansor Manan

**Affiliations:** ^1^Faculty of Pharmacy, Universiti Teknologi MARAPuncak Alam, Malaysia; ^2^Unit for Medication Outcomes Research and Education, Pharmacy, School of Medicine, University of TasmaniaHobart, TAS, Australia; ^3^Vector-borne Diseases Research Group, Pharmaceutical and Life Sciences CoRe, Universiti Teknologi MARAShah Alam, Malaysia

**Keywords:** spontaneous adverse drug reactions, pediatric, pharmacovigilance, system organ class, patient safety, medication safety, allergic skin reaction

## Abstract

Spontaneous adverse drug reactions (ADRs) reporting is a useful source of drug safety information in infants as only adult patients are routinely tested in clinical trials. This study was aimed to evaluate the spontaneously reported ADRs using WHO Adverse Reaction Terminology and to identify the common drugs associated with ADRs in children under 2 years of age. A retrospective analysis of ADR data for children below 2 years old from 2000 to 2013 was conducted using the data extracted from Malaysia’s national pharmacovigilance database, QUEST2 System. From 2000 to 2013, Malaysia’s National Pharmaceutical Control Bureau received a total of 11,932 reports for children from various healthcare facilities in Malaysia. 14.0% (*n* = 1667) of the ADRs reported for those children were related to children under 2 years old. The data retrieved was analyzed in terms of age, gender, source of reporting, type of reporters, suspected medicines and characteristics of ADRs (category, onset, severity, and outcomes). A total of 1312 ADRs reported in 907 ADR reports were analyzed. The most common ADRs reported were skin appendage disorders (60.1%), and the most frequently reported symptoms were rash (*n* = 215), maculopapular rash (*n* = 206), urticaria (*n* = 169), erythematous rash (*n* = 76), and pruritus (*n* = 58). In general, drugs from antibacterials for systemic use (58.8%) appeared to be the most common contributors to ADRs in children below 2 years old. Penicillins and other β-Lactam Antibacterials accounted for more than 40% of all drugs implicated in ADRs. The majority of ADRs were subacute reactions that occurred within 24 h of exposure to the drug. A high proportion of ADRs was classified as mild, and most victims had no sequela. Only one fatality was seen. There were 10 cases for each symptom, namely erythema multiforme and Stevens–Johnson Syndrome, observed in this study. A large proportion of ADRs in children under 2 years old were mainly caused by drugs from antibacterial for systemic use, with most of the ADRs manifesting in skin reactions. This study also reveals rare cutaneous ADRs experienced by Malaysian children under the age of 2, which constitutes a crucial cause of harm among children.

## Introduction

Drug safety in children is major health concern as systematic reviews have shown a high reporting of adverse drug reactions (ADRs) in children. Almost one in ten children in hospital will experience an ADR, 12% of which are serious (Clavenna and Bonati, 2009). Children are more vulnerable than adults as their physiological characteristics are still developing. The pharmacokinetic and pharmacodynamic parameters in children, particularly neonates and infants, change continuously due to physical changes (height and weight) and maturation of renal functions and enzyme systems (Anderson, 2002).

Randomized controlled trials (RCTs) are less effective in detecting ADRs and offer limited safety information, as certain vulnerable populations such as pediatric and geriatric patients are often underrepresented in such trials (Smyth and Weindling, 1999; Nor Aripin et al., 2012). Normally, only mild, moderate or non-serious ADRs tend to be captured during the development phase of medicines (Aagaard et al., 2008), whereas the serious and latent ones may not be captured. In addition, since RCTs are normally conducted in a controlled environment with predetermined patient criteria, the severity and staging of the disease as well as comorbidities may not reflect those found in routine clinical care, even in the relevant disease. In order to overcome the shortfalls of RCTs, spontaneous ADR reporting is a useful source of drug safety information in populations not routinely tested in RCTs, such as neonates and infants.

A large variety of drugs are prescribed to pediatric patients and an increased risk of ADRs associated with off-label prescribing particularly in children under 2 have been reported (Aagaard and Hansen, 2011; Bellis et al., 2014).

Menniti-Ippolito et al. (2000) conducted a prospective active monitoring system of ADR in children collected from family pediatricians in Italy. They concluded that a much higher risk of developing ADRs in neonate below 1 year old (34.1 per 1000 children) vs. children between 7 and 14 years old (7.4 per 1000 children) (Menniti-Ippolito et al., 2000). Moore et al. (2002) evaluated the ADR reports submitted to the US Food and Drug Administration submitted between November 1997 and December 2000 and they found that out of the 243 fatalities associated with drug therapy annually, 41 and 84% of the cases occurred during the 1st month of life and the 1st year, respectively. Meanwhile, Kaguelidou et al. (2016) evaluated ADR reports of neonates (less than 1 month of life) registered in the pharmacovigilance database from 1986 to 2012 in France. More than half of the ADR reports (*n* = 995) were classified as serious with a median age of the included patients of 9 days (Kaguelidou et al., 2016). Although studies have been conducted on the ADRs in children reported to international and Malaysian databases, only brief information on the characteristics of ADRs and drugs in neonates and infants were reported, particularly among children under 2 years of age (Aagaard et al., 2010b; Star et al., 2011; Wallerstedt et al., 2011; Aldea et al., 2012; Barzaga Arencibia et al., 2012). Our previous paper described in general the ADRs reported in respect to the Malaysian pediatric population (from birth to 17 years of age) (Rosli et al., 2016). Thus, the aim of this study is to characterize the ADRs for children under 2 and to identify the common drugs associated with ADRs. The current analysis complements our previous paper and provides an insight into the incidence and type of ADRs in Malaysian neonates and infants by analyzing ADRs for children reported to the Malaysian National Centre for Adverse Drug Reaction. Factors connected to age, gender, type of reporters, common therapeutic groups, category of ADRs, time to onset, intensity and outcomes of ADRs were also evaluated.

## Materials and Methods

### Setting

This retrospective study was carried out at National Centre for ADR Monitoring at the National Pharmaceutical Control Bureau (NPCB) using ADR data from the national pharmacovigilance database, QUEST2 System. The QUEST2 system contains extensive data of spontaneous ADR reports in Malaysia including those reported by healthcare professionals in government and/or private health facilities, other health care professionals, pharmaceutical companies and consumers.

Every ADR reports submitted to the NPCB must include the following information: patient’s particulars, the suspected medicine(s), the presumed ADR(s), and the reporter’s details. Upon receipt of ADR reports, the information is assessed by trained staff at the NPCB and all findings in each report, including the causality classification, are discussed in the Malaysian Adverse Drug Reactions Advisory Committee (MADRAC) meeting prior to submission to Malaysia’s Drug Control Authority (DCA) and the WHO Collaborating Centre in Uppsala (Uppsala Monitoring Centre; WHO-UMC).

For causality assessment, MADRAC utilizes the WHO-UMC Causality Assessment. The causality between the suspected drug/s and the reaction/s is assessed as certain, probable, possible, unlikely, unclassified or unclassifiable. The individual categories of causality are presented in Supporting Information 1 (SI 1).

In the data retrieved from the QUEST2 system, an ADR registration number was assigned automatically upon entry into the database system for record identification. No informed consent was obtained prior to analysis as the patient information was already anonymized and de-identified when the data was entered into the database system.

Approval to conduct this study was obtained from the National Institute of Health (NIH) and Medical Research and Ethics Committee (MREC) in the Ministry of Health, prior to implementation of the study (NMRR-14-1231-21610). Permission to access to the QUEST2 database was also granted.

### Data Extraction

All ADR reports on children under 2 years of age reported in the QUEST system from 2000 to 2013 were included in this study. Data on patient’s details (age and gender), type of reporters, active substance of the medicine, and characteristics of ADRs (category, time to onset, severity and outcomes) were extracted from the QUEST2 ADR database into Microsoft^®^ Excel files. Onset of ADRs was categorized as: acute (occurring within 60 min); subacute (occurring within 1–24 h); or latent (occurring after 2 days). Onset can be defined as the time from the start of drug administration to the onset of a reaction. Severity is defined as the intensity of a specific ADR event and here is categorized as: mild; moderate; severe; or fatal. The active substances of the medicines were classified according to Anatomical Therapeutic Chemical Classification (ATC). The medicines about which the ADRs were reported are presented at ATC level 3 to present the large amount of data in a comprehensive way.

The material comprised all ADR reports on children from birth to 2 years of age reported to the QUEST system from 2000 to 2013. These ADR reports were then subdivided according to the age categories based on the International Conference on Harmonization Guidelines on Clinical Investigation of Medicinal Products in the Pediatric Population: neonates ≤ 27 days and infants ≤ 23 months.

The ADRs are grouped and presented according to the WHO Adverse Reaction Terminology (WHO-ART) system organ classes (SOCs). For ADRs reporting to WHO-UMC, all national pharmacovigilance centres may either use WHO-ART or Medical Dictionary for Regulatory Activities (MedDRA) terms or codes for reporting to the WHO Global ICSR Database System, Vigibase. Since, the National Centre for ADR Monitoring in Malaysia has been using WHO-ART for SOC classification in their reporting to UMC, the same classification for SOC (WHO-ART) was used in this study. We counted each report as a different case with records of more than one term belonging to the same SOC.

### Reports Excluded

In order to enhance the quality of data included in this study, only ADRs with causality assessed as certain, probable/likely and possible were analyzed. As one of the National Centers participating in the WHO Programme for International Drug Monitoring, NPCB utilized WHO-UMC causality assessment system for evaluation of ADR reports in Malaysia. ADR reports relating to non-Malaysian citizens and those that had not been reviewed by the MADRAC were excluded from the study, as were reports with age and onset of ADRs not specified. Other ADR reports that were excluded were reports on food supplements, traditional and veterinary products, along with ADRs induced by drug administration to mothers.

### Statistics

Data on ADRs according to gender, type of reporters, therapeutic groups and category of ADRs were described as frequencies and percentages. The analysis was undertaken using Microsoft Excel 2013 and IBM SPSS Version 19.

## Results

### Characterization of ADR Reports

From 2000 to 2013, NPCB received a total of 11,932 reports on children from various healthcare facilities in Malaysia. Of that volume, approximately 14.0% (*n* = 1667) of the ADRs reported were related to children under 2 years old.

**FIGURE 1:**
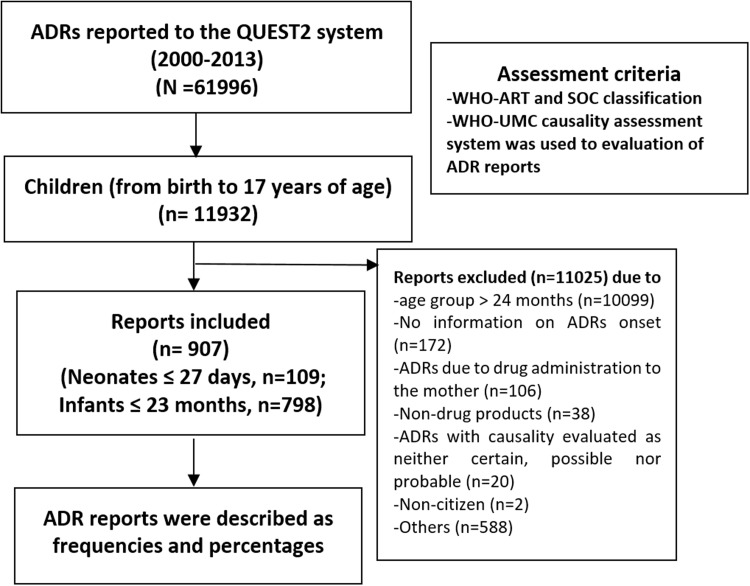
**Flow chart of methodology and adverse drug reaction (ADR) reports included in the present study**.

The following reports were excluded from the study: Age group > 2 years old (*n* = 10099); ADRs with causality evaluated as neither certain, possible nor probable (*n* = 20); ADRs due to drug administration to the mother (*n* = 106); non-citizen (*n* = 2); non-drug products (*n* = 38); reports with no information on ADR onset (*n* = 172); and miscellaneous/others (*n* = 588). After exclusion criteria, only 907 ADR reports (neonates, *n* = 109; infants, *n* = 798) remained for analysis (see **Figure 1**).

In the reports where the gender was known, 57.2% (*n* = 519) concerned boys. More than 90% of the reports were sent by healthcare providers in government health facilities. Most of the reports were sent by pharmacists (*n* = 466, 51.4%), followed by doctors (*n* = 351, 38.7%). The majority of the reports (*n* = 610, 67.3%) document one ADR per report, followed by two ADRs per report (*n* = 210, 23.2%) and three ADRs per report (*n* = 71, 7.8%).

### System Organ Class

**Table 1** illustrates the System Organ Class for ADR reports received for children under 2 years old. From 907 ADR reports, there were 1312 types of ADRs reported, mainly Skin Appendage Disorders (60.1%) and Body as a Whole – General Disorders (10.1%). Other ADRs reported were Gastrointestinal System Disorders (6.0%) and Respiratory System Disorders (5.1%). The most common preferred terms reported were rash (*n* = 215), maculopapular rash (*n* = 206), urticaria (*n* = 169), erythematous rash (*n* = 76), and pruritus (*n* = 58).

**Table 1:** System organ class for children below 2 years old.

System organ class	Neonates	Infants	Total
Skin and Appendages Disorders	60	729	789
Body as a Whole General Disorders	8	124	132
Gastrointestinal System Disorders	8	71	79
Respiratory System Disorders	12	55	67
Urinary System Disorders	12	30	42
Central and Peripheral Nervous System Disorders	8	33	41
Cardiovascular Disorders, General	10	25	35
Heart Rate and Rhythm Disorders	4	16	20
Platelet, Bleeding and Clotting Disorders	6	11	17
Liver and Biliary System Disorders	0	15	15
Neonatal and Infancy Disorders	12	3	15
Psychiatric Disorders	3	12	15
Vascular (Extracardiac) Disorders	0	13	13
Vision Disorders	0	10	10
Application Site Disorders	2	4	6
Metabolic and Nutritional Disorders	1	4	5
Endocrine Disorders	1	1	2
Red Blood Cell Disorders	1	2	3
White Cell and Reticuloendothelial system disorders	1	2	3
Reproductive Disorders, Female	0	1	1
Resistance Mechanism Disorders	1	0	1
Secondary Terms Events	1	0	1
**TOTAL**	**151**	**1161**	**1312**

### Anatomical Therapeutic Chemical Classifications

Anti-infectives for Systemic Use (61.9%) was the most common therapeutic group reported for ADRs in children below 2 years old. As shown in **Table 2**, other common therapeutic groups were for the Nervous System (15.6%), Respiratory System (6.8%), Alimentary Tract and Metabolism (4.2%), and Sensory Organs (3.9%). ADR reports related to the top three therapeutic groups were further analyzed according to onset, intensity and outcomes of ADRs.

**Table 2:** Therapeutic groups reported for ADRs in children below 2 years old.

ATC Level 1	Neonates	Infants	Total
Anti-infectives for Systemic Use	55	506	561
Nervous System	10	131	141
Respiratory System	13	43	56
Alimentary Tract and Metabolism	7	31	38
Sensory Organs	5	30	35
Cardiovascular System	9	7	16
Musculo-skeletal System	3	16	19
Blood and Blood Forming Organs	3	8	11
Dermatologicals	2	7	9
Systemic Hormonal Preparations, Excl. Sex Hormones and Insulins	0	7	7
Antineoplastic and Immunomodulating Agents	2	4	6
Various	0	5	5
Antiparasitics Products, Insecticides and Repellents	0	3	3
**TOTAL**	**109**	**798**	**907**

Antibacterials for Systemic Use contributed the highest number of ADRs among the antiinfective agents. Penicillin (30.3%) and other β-Lactam Antibacterials (13.6%) were the most frequent agents reported for ADRs. The others were: Macrolides, Lincosamides, and Streptogramins (6.0%); Aminoglycosides Antibacterials (3.5%); Other Antibacterials (3.3%); and Sulphonamides and Trimethoprim (1.7%). Antivirals for Systemic Use that involved Direct Acting Antivirals (2.3%) were also among agents that were repeatedly reported for ADRs.

For the Nervous System, the reports received were mainly related to Analgesics, namely Other Analgesics and Antipyretics (10.5%). Other common agents for the Nervous System belonged to Antiepileptics (2.9%). For the Respiratory System, Antihistamines for Systemic Use (1.5%), Adrenergics for Systemic Use (1.4%), and Other Systemic Drugs for Obstructive Airway Diseases (1.1%) were often reported for ADRs in this study.

### Onset, Intensity, and Outcomes of ADRs

The onset and the intensity of ADRs related to the three common therapeutic groups with frequently reported pharmacological groups are listed in **Table 3**. The majority of ADRs induced by Anti-infectives for Systemic Use were subacute reactions, occurring within 24 h after drug exposure, except for the Other Antibacterials group, where mainly acute reactions occurred, within 1 h of drug exposure. A high proportion of ADRs were classified as mild and moderate whilst there were a small number of severe ADRs.

**Table 3:** Onset and intensity of ADRs related to commonly reported pharmacological groups in children below 2 years old.

		Type of ADR (onset)				Type of ADR (intensity)			
Reported pharmacological group	Number of cases	Acute	Subacute	Latent	Mild	Moderate	Severe	Unknown
**ANTI-INFECTIVES FOR SYSTEMIC USE**
**Antibacterials for Systemic Use**								
β-Lactam Antibacterials, Penicillins	275	45	163	67	147	112	14	2
Other β-Lactam Antibacterials	123	18	80	25	62	46	11	4
Macrolides, Lincosamides and Streptogramins	54	5	39	10	29	22	3	0
Other Antibacterials	30	13	8	9	12	15	3	0
Aminoglycoside Antibacterials	32	8	11	13	12	14	6	0
Sulfonamides and Trimethoprim	15	1	9	5	5	8	2	0
**Antivirals for Systemic Use**								
Direct Acting Antivirals	21	5	13	3	7	12	2	0
**NERVOUS SYSTEM**
Antiepileptics	26	6	9	11	10	10	6	0
Other Analgesics and Antipyretics	95	26	58	11	50	39	4	2
**RESPIRATORY SYSTEM**
Adrenergics for Systemic Use	13	4	7	2	4	7	2	0
Antihistamines for Systemic Use	14	3	9	2	2	5	7	0
Other Systemic Drugs for Obstructive Airway Diseases	10	0	5	5	3	1	3	3
Adrenergics Inhalants	8	3	5	0	5	3	0	0

Similar to Anti-infectives for Systemic Use, the majority of ADRs related to medicines for the Nervous System and Respiratory System were subacute reactions, with a high proportion of mild and moderate ADRs. Only three drugs were continued despite ADRs and no rechallenge was performed in the majority of cases after ADRs developed.

The majority of ADRs observed in children under 2 years old did not result in sequelae. However, at the time of reporting, a high number of children with ADRs had not yet recovered with a small number of ADRs cases had recovered but with sequelae. Only one ADR had a fatal outcome.

### Common Drugs Associated with ADRs

Drugs commonly reported for ADRs in children are summarized in **Table 4**. A drug was considered common when the number of ADR reports received for that particular drug was more than 10 and only ADR SOCs with more than 10 cases were included. As shown in the table below, Antibacterials for Systemic Use such as Penicillin (*n* = 93), Ampicillin (*n* = 56), Amoxicillin (*n* = 51), Cloxacillin (*n* = 43), and Cefuroxime (*n* = 51) were often reported for ADRs in neonates and infants. Most of the ADRs associated with these drugs were skin reactions. Other frequently reported drugs were Paracetamol (*n* = 93), which induced skin reactions, and Cyclopentolate/Phenylephrine (*n* = 19) which induced apnoea.

**Table 4:** Common drugs associated with ADRs in children below 2 years old.

Common drugs	ADR reports	Most common preferred terms
**J01 Antibacterials for Systemic Use**
*J01C β-Lactam Antibacterials, Penicillins*
Penicillin	93	Rash Maculopapular (*n* = 36), Rash (*n* = 25), Urticaria (*n* = 21), Rash Erythematous (*n* = 13), Pruritus (*n* = 12)
Ampicillin	56	Rash (*n* = 16), Rash Maculopapular (*n* = 18), Urticaria (*n* = 15)
Amoxicillin	51	Rash (*n* = 17), Rash Maculopapular (*n* = 15), Urticaria (*n* = 12)
Cloxacillin	43	Rash Maculopapular (*n* = 14), Rash (*n* = 12)
*J01D Other β-Lactam Antibacterials*
Cefuroxime	51	Rash (*n* = 18), Urticaria (*n* = 15), Rash Maculopapular (*n* = 10)
*J01F Macrolides, Lincosamides and Streptogramins*
Erythromycin	39	Rash Maculopapular (*n* = 12), Rash (*n* = 10)
**N02 Analgesics**
*N02B Other Analgesics and Antipyretics*
Paracetamol	93	Rash (*n* = 31), Urticaria (*n* = 19), Rash Maculopapular (*n* = 14)
**S01 Opthalmologicals**
*S01F Mydriatics and Cycloplegics*
Cyclopentolate/Phenylephrine	19	Apnoea (*n* = 12)

### Cutaneous ADRs Reported in Children

In this study, the cutaneous ADRs that were frequently reported were erythema multiforme (*n* = 10) and Stevens–Johnson Syndrome (*n* = 10). For erythema multiforme, amoxicillin (*n* = 3), erythromycin (*n* = 2) and paracetamol (*n* = 2) were among the contributing agents, whilst for Stevens–Johnson Syndrome, amoxicillin/clavulanate (n = 3), carbamazepine (*n* = 3), and sulfamethoxazole/trimoxazole (*n* = 2) were among the drugs reported. The lists of selected cutaneous ADRs detected in this study are summarized in **Table 5**.

**Table 5:** Selected cutaneous ADRs reported in children below 2 years old.

SOC (WHO-ART CLASSIFICATION)	TOTAL	SUSPECTED DRUGS
Erythema Multiforme	10	Amoxicillin (*n* = 3), Ampicillin (*n* = 1), Cloxacillin (*n* = 1), Erythromycin (*n* = 2), Paracetamol (*n* = 2) Penicillin (*n* = 1)
Steven–Johnson Syndrome	10	Amoxycilin/clavulanate (*n* = 3), Carbamazepine (*n* = 3), Morphine (*n* = 1), Phenobarbitone (*n* = 1), sulfamethoxazole/Trimethoprim (*n* = 2),
Fixed Drug Eruption	1	Paracetamol (*n* = 1)
Epidermal Necrolysis	1	Amoxicillin/Clavulanate (*n* = 1)

## Discussion

Previous studies have confirmed that neonates and infants have a higher risk of developing ADRs than other populations (Menniti-Ippolito et al., 2000; Clavenna and Bonati, 2009; Alomar, 2014). This current study revealed a low reporting of ADRs in this age group. This is probably due to under-reporting, as the proportion of ADRs for Malaysian children under 2 years of age was lower than that reported to WHO VigiBase (Rosli et al., 2016).

Apart from age, being female has been postulated as one of the risk factors of developing ADRs (Smyth et al., 2012). The biological differences in term of anatomy and physiology in males and females significantly affects the way the body deals with drugs by altering their pharmacokinetics and pharmacodynamics (Alomar, 2014). A systematic review of ADRs in children discovered that out of 19 studies evaluated, 10 considered gender as a risk factor and females were more likely to have an ADR (Smyth et al., 2012). On the other hand, a multicentre cohort study involving hospitalized pediatrics from general medical ward from hospitals in five countries, including Malaysia, discovered that gender was not significantly associated with ADRs (Rashed et al., 2012b). The same findings were reported by other studies, which reported a similar proportion of ADR reports for males and females. Even so, in these studies there was no information divided by gender on the proportion of ADRs for specific age groups, namely neonates and infants (Aagaard et al., 2010a,b; Aldea et al., 2012). In the current study, the higher proportion of ADRs in males were most likely contributed by the higher crude birth rate per 1000 population for males than for females in Malaysia during the study period (Ministry of Health Malaysia, 2012, 2015).

In Malaysia, ADR reporting is mainly done by healthcare personnel from government health facilities, with pharmacists (51.4%) being the main reporters. This is in agreement with a study by Le et al. (2006). Our current study reveals that pharmacists in Malaysia are actively reporting ADRs, which is different in Denmark (Aagaard et al., 2010a,b). The high number of reports submitted by pharmacists are in tandem with the increase in pharmacists working in public hospitals since 2009, because the Malaysian Ministry of Health introduced a requirement for pharmacists to complete stipulated years of compulsory service in public sector before they can register with the Pharmacy Board of Malaysia (Hadi and Ming, 2011). Besides setting ADR reporting as one of the main key performance indicators in the Ministry, the initiatives taken by the NPCB in promoting ADRs reporting in Malaysia has successfully boosted the number of ADRs being reported. The Pharmaceutical Services Division at the Ministry of Health, which is responsible for planning and controlling all activities related to pharmacy services in Malaysia, has incorporated ADR reporting in the pharmacist training modules. Every pharmacy candidate completes these modules during their compulsory training to become a registered pharmacist. However, while these initiatives have positively improved the ADR reporting culture in the government health facilities, it has only had a minimal effect on the private health facilities, as a very low number of ADR reports are received from these latter facilities (Rosli et al., 2016). Meanwhile, studies have shown that approximately 60% of the participating general practitioners have unsatisfactory knowledge on how to report an ADR (Agarwal et al., 2013). The same study also pointed out that, at a result of not knowing how to submit an ADR report, up to 73% of the participating general practitioners reported an unsatisfactory attitude toward ADR reporting (Agarwal et al., 2013).

In the general child population, as documented by other studies, the largest share of ADR SOCs reported was Skin Appendage Disorders (60.1%) (Morales-Olivas et al., 2000; Clavenna and Bonati, 2009; Star et al., 2011; Gallo et al., 2012; Rashed et al., 2012a). A study by Star et al. (2011) found that ADRs related to skin reactions were more frequently seen in children than in adults. One of the underlying causes is the skin physiology of children, especially neonates and infants, as humans are born with partially mature and functionally developing epidermises. Consequently, the epidermal barrier function is compromised, leaving the skin more permeable and vulnerable to chemical and microbial aggressions and skin disease in the first 2 years of someone’s life (Stamatas et al., 2011).

In general, the therapeutic groups that have been linked to ADRs were not significantly different among studies conducted in children (Morales-Olivas et al., 2000; Aagaard et al., 2010b; Star et al., 2011; Aldea et al., 2012). Nevertheless, the sub-groups were different due to changes in prescribing practices for some therapeutic groups over time and distinct drug prescribing trends among countries or regions (Aagaard et al., 2010a). In this study, it has been shown that Antibacterials for Systemic Use were the most common agents reported for ADRs. In a multicenter prospective cohort study of hospitalized children involving five countries, including Malaysia, similar findings were observed by Rashed et al. (2012a). A huge number of Antibacterials for Systemic Use were prescribed in the general pediatric population and a higher number of ADRs were reported for agents from this therapeutic group (Clavenna and Bonati, 2009; Priyadharsini et al., 2011; Barzaga Arencibia et al., 2012; Gallo et al., 2012; Rashed et al., 2012a). Due to the issue of antibiotic resistance, often multiple types of antibiotics and broad spectrum antibiotics at high doses were used for neonatal infection (Manan et al., 2016). In the current study, the most commonly involved antibiotics in the reported ADRs were β-lactam and Macrolides, which is consistent with findings reported in Italy (Menniti-Ippolito et al., 2000) and Singapore (Kidon and See, 2004). These antibiotics were associated with ADRs related to skin reactions, which is also in parallel with the findings of other studies reporting ADRs from the national pharmacovigilance databases in Denmark (Aagaard et al., 2010b), India (Priyadharsini et al., 2011), and Iran (Khotaei et al., 2008). This study and other studies have shown that ADRs resulting from the use of β-lactam antibiotics are usually mild, with a small proportion of severe reactions (Morales-Olivas et al., 2000; Clarkson and Choonara, 2002; Moore et al., 2002; Rashed et al., 2012a; Li et al., 2014). The ADRs observed in children were mild, and the majority of the ADRs did not lead to sequelae.

Other than antibiotics, mydriatic eye drop (cyclopentolate and phenylephrine) is among drugs that are commonly reported for ADRs. Cyclopentolate and phenylephrine eye drops are rarely absorbed to systemic circulation but serious side effect like apnoea, tachycardia, rash, feeding intolerance, discomfort, gastric dilatation, and ileus has been reported in some cases (Knight, 1994; Sarici et al., 2001; Ozgun et al., 2014). Mydriatic eye drops are normally applied prior to routine eye examination for retinopathy of prematurity (ROP) screening in premature infants. Early detection of ROP is deemed crucial as it can leads to vision impairment and blindness if left untreated (Fierson et al., 2013). In this study, 12 cases of apnoea were observed after application of these agents.

Although hypersensitivity reactions are rare in infants, they do happen in this population, normally manifested as cutaneous ADRs (Carder, 2005). It is already known that systemic antibiotics and anticonvulsants are the major cause of cutaneous ADRs (Morales-Olivas et al., 2000; Choon and Lai, 2012; Diaz and Ciurea, 2012; Lin et al., 2014). In this study, there were 10 cases of erythema multiforme induced by amoxicillin, erythromycin and paracetamol. Erythema multiforme is a reactive eruption on the skin and mucous membranes characterized by well-demarcated, targetoid, erythematous plaques or patches that develop a dusky or bullous center within several days. The majority of erythema multiforme cases have been reported with multiple drugs including β-lactam, macrolides, aminoglycosides, glycopeptides and other antibiotics (Diaz and Ciurea, 2012; Sokumbi and Wetter, 2012). For paracetamol, cutaneous adverse reactions to standard dosages are quite rare and only a few cases of erythema multiforme have been reported with the use of paracetamol. In the current study only two cases of erythema multiforme related to paracetamol were reported.

On the other hand, there were ten cases of Stevens–Johnson Syndrome seen in this study. This ADR has been reported with amoxicillin/clavulanate, carbamazepine and sulfamethoxazole/trimoxazole. Stevens-Johnson Syndrome is a serious cutaneous eruption defined by extensive exfoliation and mucosal membrane involvement. Like erythema multiforme, Stevens–Johnson Syndrome can be induced by many drugs, such as sulfamethoxazole/trimethoprim and other sulphonamide antibiotics, aminopenicillins, cephalosporins, quinolones, carbamazepine, phenytoin, and phenobarbital (Harr and French, 2010; Diaz and Ciurea, 2012).

This study presents the ADR data from the QUEST2 pharmacovigilance database reported for Malaysian children under 2 years old and only reports the reporting of ADRs in children, characteristics of ADRs (category, onset, intensity, outcomes), and the most frequently reported drugs with their common clinical presentation of ADRs. In this study, detailed management of the reaction was not studied since some of this data were not readily available. Furthermore, the relationship between the use of off-labeled drugs and the occurrence of ADRs was not studied since the majority of the ADR reporting did not indicate that.

Current literature indicated a relatively sparse spontaneous reporting of pediatric ADRs in Malaysia. Active surveillance studies should be conducted in the Malaysian pediatric population since our findings as well as studies reported by Rashed et al. (2012a,b) have shown relatively high rates of ADRs experienced by pediatric inpatients in Malaysia. Future studies monitoring ADRs should be undertaken in a real hospital setting involving neonates and children under 2 years of age, as this study observed only a small number of ADRs for this group being reported to the national pharmacovigilance system. It is vital as other literature reported prominent ADR reporting in this population.

The data is derived entirely from the Malaysian ADR spontaneous reporting database. Underreporting of ADRs is one of the main limitations of this system, as proven by a review which discovered a lower ADR reported from studies using ADR data from the pharmacovigilance database than studies using a chart review (Hazell and Shakir, 2006; Aagaard et al., 2010a).

## Conclusion

The data from the current study reveals that ADRs in children under 2 years old are mainly related to antibiotics, which could be a reflection of the common usage of antibiotics in this population group.

A large proportion of ADRs in children under 2 years old was mainly induced by drugs from the antibacterial for systemic use group, and most of the ADRs were skin reactions. This study also reveals rare cutaneous ADRs experienced by Malaysian children younger than 2 years old, which constitutes a crucial cause of harm in children.

## Author Contributions

Conceived and designed the experiments: RR, NA, LM, and MM. Performed the experiments: RR. Analyzed the data: RR, AD, NA, LM, and MM. Contributed reagents/materials/analysis tools: NA, LM, and MM. Wrote the paper: RR, AD, NA, LM, and MM.

## Conflict of Interest Statement

The authors declare that the research was conducted in the absence of any commercial or financial relationships that could be construed as a potential conflict of interest.
