# Supplementary material for: Reported Adverse Drug Reactions in Infants: A Nationwide Analysis in Malaysia
Source: Front Pharmacol. 2017 Feb 10;8:30. doi: 10.3389/fphar.2017.00030 (PMC5300992; doi:10.3389/fphar.2017.00030)
Supplement: Supplementary file 1 [file Data_Sheet_1.DOCX]

**SI 1. The categories of ADR causality assessment**

Reference: WHO-Uppsala Monitoring Centre. The use of the WHO-UMC system for standardised case causality assessment. Available online: http://who-umc.org/Graphics/24734.pdf. Accessed 8 Aug 2016*.*

**Certain**

*A clinical event, including a laboratory test abnormality, that occurs in a plausible time relation to drug administration, and which cannot be explained by concurrent disease or other drugs or chemicals. The response to withdrawal of the drug (dechallenge) should be clinically plausible. The event must be definitive pharmacologically or phenomenologically, using a satisfactory rechallenge procedure if necessary*

**Probable/likely**

*A clinical event, including a laboratory test abnormality, with a reasonable time relation to administration of the drug, unlikely to be attributed to concurrent disease or other drugs or chemicals, and which follows a clinically reasonable response on withdrawal (dechallenge). Rechallenge information is not required to fulfil this definition*

**Possible**

*A clinical event, including a laboratory test abnormality, with a reasonable time relation to administration of the drug, but which could also be explained by concurrent disease or other drugs or chemicals. Information on drug withdrawal may be lacking or unclear.*

**Unlikely**

*A clinical event, including a laboratory test abnormality, with a temporal relation to administration of the drug, which makes a causal relation improbable, and in which other drugs, chemicals, or underlying disease provide plausible explanations*

**Conditional/unclassified**

*A clinical event, including a laboratory test abnormality, reported as an adverse reaction, about which more data are essential for a proper assessment or the additional data are being examined*

**Unassessable/unclassifiable**

*A report suggesting an adverse reaction that cannot be judged, because information is insufficient or contradictory and cannot be supplemented or verified*
